# Amphiphilic Molecules, Interfaces and Colloids

**DOI:** 10.3390/molecules30010025

**Published:** 2024-12-25

**Authors:** Khristo Khristov, Plamen Tchoukov

**Affiliations:** Institute of Physical Chemistry, Bulgarian Academy of Sciences, “Acad. G. Bonchev” Str. Bl. 11, 1113 Sofia, Bulgaria

## 1. Introduction

Colloids, such as emulsions, foams, sols and gels, play integral roles in living organisms, the natural environment, resource extraction, pharmaceuticals, cleaning products, processing industries and almost all aspects of our everyday lives [1]. Generally, colloid systems consist of a continuous phase and a dispersed phase, which is in the form of finely distributed particles with sizes in the 1 nm–1000 nm range. Such a high degree of dispersity is inevitably related to a very large interface separating the two phases. Thus, the interfacial properties and related surface forces greatly determine the properties of the colloid system as a whole [2,3].

Amphiphilic molecules contain structural groups with affinity for either “water” or “oil”, two immiscible solvents. Their amphiphilic character determines preferential adsorption at the interface and enables self-assembly into various structures, such as micelles, vesicles, monolayer, bilayers and lamellar phases in the bulk [4]. These self-assembled structures play essential roles in modifying and controlling the functionality of the colloid systems. Selecting amphiphiles with appropriate structures and properties is critical for designing functional colloids for specific applications.

In light of growing environmental awareness, there is increased interest in using “green” surfactants that are derived from sustainable sources. The aim is to replace conventional surfactants with alternatives that are biodegradable, non-toxic and produced through sustainable processes with a reduced carbon footprint [5]. Developing new green surfactants requires in depth understanding of the relationship between a molecule’s chemical structure and its physicochemical properties.

This Special Issue (SI) attracted a diverse collection of research that highlights the important relationship between the molecular structure of amphiphiles, their interfacial behavior and the properties of the corresponding colloidal systems. This SI includes research topics ranging from fundamental thermodynamic investigations to the fabrication of novel materials with tailored properties.

## 2. An Overview of the Contributions

Peychev et al. (Contribution 1) present an improved approach for more accurate experimental determination of the free energy of transfer of a fluorocarbon -CF_2_- group from oil to water. This determination is based on adsorption data of fluorosurfactants at the water/hexane interface. Knowledge of this thermodynamic parameter is essential for predicting the partitioning of fluorinated surfactants between water and oil, as well as between water and lipid membranes, and for evaluating their environmental impact.

Wei et al. (Contribution 2) report the fabrication of supramolecular self-assembled organohydrogels mixing double-tailed zwitterionic quaternary ammonium amphiphiles and phosphomolybdic acid in a binary solvent of water and dimethyl sulfoxide.

Lu et al. (Contribution 3) explored the effect of the orientations of the ester group of two series of newly synthesized methyl d-glycopyranoside-based esters on their physicochemical properties. The observed trends in solubility and adsorption, and the comparison with the commercial octyl d-glycosides, provide clear guidance on how the structure of molecules affects their properties.

Guo et al. (Contribution 4) entertained the idea of creating a protocell model in water using sodiummonododecyl phosphate (SDP), isopentenol (IPN) and pyrite (FeS_2_) mineral particles. The FeS_2_ particles promote the IPN/SDP vesicles’ fusion and growth and influence their morphological evolution. This research is thought, in the long run, to shed light on the possible model systems for protocell membranes.

Gutiérrez-Fernández et al. (Contribution 5) report a new dispersive solid-phase extraction of melatonin using graphene mixtures with sepiolite and bentonite clays as sorbents combined with fluorescence detection. The method is applied to herbal samples containing melatonin.

In the study by Wang et al. (Contribution 6), double-chain lactobionic amide quaternary ammonium salts were synthesized via the amidation of lactobionic acid with N,N-dimethyldipropyltriamine to obtain glycosylamides, followed by quaternization with bromoalkanes of different chain lengths. The resulting product is a novel glucosamine-based cationic surfactant characterized by low foaming, antibacterial properties, antistatic properties, salt resistance, and the ability to form stable vesicular systems. It shows potential for applications in diverse fields, including drug delivery systems, biomimetic membranes, microreactors, specialty chemicals and the food industry.

Liu et al. (Contribution 7) studied liquid droplets and liquid marbles (droplets of 1M Fe_2_Cl aqueous solution fully coated with hydrophobic polyvinylidene fluoride powder) at the liquid–liquid interface to investigate capillary forces and interactions at intra- and inter-cellular scales. Interfacial liquid marbles possess unique internal and external features, making them a promising model and a versatile tool for the investigation of different interfacial phenomena.

Kamburova et al. (Contribution 8) investigated the kinetics of amyloid aggregation indirectly by monitoring the changes in the polydispersity of a mixed dispersion of amyloid β peptide (1–40) and composite liposomes as a function of temperature and pH. Despite homotaurine’s specific bioactivity in natural cell membranes, this study highlights its additional inhibitory effect on amyloid peptide aggregation, attributed to charge interactions and ‘molecular crowding’.

Alvarez et al. (Contribution 9) studied the aggregation behaviors of four amino acid-based surfactants in the presence of five linear diamine counterions. Electrical conductimetry was used to determine the CMCs for each system, and dynamic light scattering was used to evaluate the micellar size. The obtained CMCs correlated with each surfactant’s partitioning coefficients, logP (water/octanol) value. The obtained results highlight the relationship between the structure of the surfactants and their physicochemical properties.

Chunlin Xu et al. (Contribution 10) fabricated a novel single-tailed dynamic covalent surfactant 1-methyl-3-(2-(4-((tetradecylimino)methyl) phenoxy)ethyl)-3-imidazolium bromide (C_14_PMimBr). In aqueous solutions with increasing concentrations, C_14_PMimBr forms micelles, vesicles and hydrogels. The authors envision the application of their approach to the design of novel stimuli-responsive surfactant systems for drug delivery and targeted drug release.

The review paper “Law and Order of Colloidal Tectonics: From Molecules to Self-Assembled Colloids” authored by Leclercq (Contribution 11) discusses in depth the emerging concept of colloidal tectonics. Tectons are molecular building blocks capable of spontaneously forming supra-colloidal structures. The author presents colloidal tectonics as a phenomenon that bridges the gap between the soft self-assemblies of small amphiphilic molecules, driven by hydrophobic interactions (such as micelles or vesicles), and rigid crystalline structures with periodic molecular arrangements. The review refers to examples from biotic systems and theoretical insights as a potential guide for self-assembled systems with applications in drug delivery, catalysis, and other applications.
